# Novel Sustained-Release of Propafenone through Pellets: Preparation and *in Vitro*/*in Vivo* Evaluation

**DOI:** 10.3390/ijms150915503

**Published:** 2014-09-02

**Authors:** Li Zhang, Ping Jiang, Ji Liu

**Affiliations:** 1 Emergency Department, Shanghai Pulmonary Hospital, Tongji University School of Medicine, 507 Zheng Min Rd., Yangpu District, Shanghai 200433, China; E-Mails: pangzilama@sina.com (L.Z.); jiangpingpj@yeah.net (P.J.); 2 Department of Anesthesia, Shanghai Pulmonary Hospital, Tongji University School of Medicine, 507 Zheng Min Rd., Yangpu District, Shanghai 200433, China

**Keywords:** propafenone, pellets, sustained-release, pharmacokinetic, extrusion–spheronization method

## Abstract

In this study, an extrusion-spheronization method was applied successfully to fabricate propafenone hydrochloride (PPF) sustained-release pellets. Using scanning electron microscopy, it was shown that the PPF pellets had a mean size of approximately 950 µm with a spherical shape. The *in vitro* release profiles indicated that the release of PPF from the pellets exhibited a sustained release behavior. The relatively high correlation coefficient (*r*) values obtained from the analysis of the amount of the drug released *versus* the square root of time indicated that the release followed a zero order kinetic model. A similar phenomenon was also observed in a pharmacokinetic study in dogs, in which the area under the curve (AUC) of the pellet formulation was 1.2-fold higher than that of PPF tablets. The present work demonstrated the feasibility of controlled delivery of PPF utilizing microcrystalline cellulose (MCC)-based pellets.

## 1. Introduction

**Figure 1:**
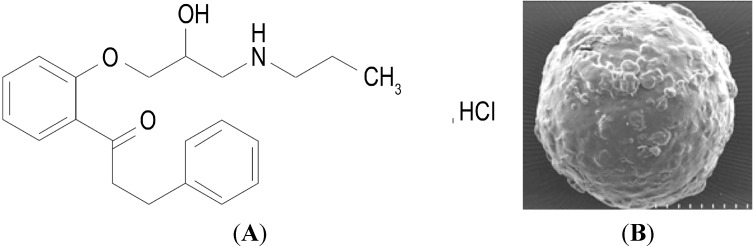
The structure of propafenone hydrochloride (**A**); and transmission electron microscope photograph of propafenone hydrochloride (PPF) (**B**). Magnification: 5000×, Scale bar = 500 µm.

Propafenone hydrochloride (PPF, Figure 1), a class IC antiarrhythmic drug (sodium channel inhibitor), has been widely used as a treatment for ventricular and supraventricular arrhythmias since it was approved in Europe and the United States in the 1980s [1]. The initial marketed preparation was rapidly absorbed from the gut when given by mouth and was absorbed rapidly in the gastrointestinal tract (GI) [2]. It has high first pass effect with a mean bioavailability about 4.8%–23.5% and is metabolized by the liver [3]. The half life of PPF is about 3–4 h [3]. Some scholars have observed that adverse reactions of PPF treatment take place when PPF reached the peak plasma concentration. Thus, small doses of multiple administration were suggested [4]; it was recommended to be taken 4–6 times daily [4].

Different formulation approaches such as sustained-release tablets [5,6,7] and osmotic tablets [8] have been developed to sustain the release of drug and improve patient compliance. However, the above formulations may need complex technology, have high cost and were unable to meet industrial production. In addition, there is no report in the literature on the application of a conventional pelletisation technique for preparation of PPF pellets for an oral delivery system in order to increase patient compliance through a reduction of dosing per day, and to reduce inter- and intra-individual variations in bioavailability.

Pellets are spherical, free-flowing granules with a narrow size distribution of 0.5–1.5 mm [9]. Designing and developing dosage form flexibility, reduction of variability of drug dissolution and plasma profiles, low friability and uniform packing characteristics, improvement of the drug safety and efficacy and spreading of drug freely throughout the contents of the GI are some desired advantages of pellets [10,11]. An attractive method for preparing pellets in the pharmaceutical industry is extrusion/spheronisation technique, which is a simple, fast and popular process with an easy scale-up capability. Production of uniform size pellets with high drug loading capacity is the major goal of the extrusion/spheronisation technique [12,13]. For preparation of pellets by this method first of all, the uniform powder mixture of drug and excipient(s) are wetted by the addition of a liquid binder, then the wet mass is pressed through an extrusion to form cylindrical extrudates, that are broken into smaller rods and immediately transformed into spherical pellets by means of a fast-rotating plate (spheroniser) and finally dried [14].

In the present study, a novel extrusion-spheronization method was employed to prepare pellets of PPF by using microcrystalline cellulose (MCC) and Eudragit S100. The drug content uniformity, physical testing, and *in vitro* drug release were investigated and the mechanism of drug release was also evaluated. In addition, the pharmacokinetic profiles of novel PPF sustained-release pellets was compared with that of commercial PPF tablets in beagle dogs.

## 2. Results and Discussion

### 2.1. Characteristics of the Pellets

Microcrystalline cellulose (MCC), a gold standard in the production of pellets, provides the proper rheological properties and cohesiveness to the moistened mass and so it is used in most formulations prepared via extrusion–spheronisation technique [15]. Moreover, MCC has a large surface area and high internal porosity and hence it can absorb and retain a large quantity of water that facilitates the extrusion process, provides plasticity to the moistened mass and enhances spheronisation [16]. In this study, pellets with MCC amount of 67.5% were found to be optimum in the formation of indented short-segment extrudate and spherical shape pellets. However, a lower amount of MCC content, under 50%, led to the production of pellets with poor physical characteristics. On the other hand, by increasing the content of MCC in the formulation, the powder mass absorbed more water content resulted in the production of more cohesive moistened mass, agglomeration of small particles and in turn larger micropellets during the pelletisation process. The amount of lactose did not have a major impact on pellet production.

The average loaded drug percent in pellets was 93.9%. The friability of the pellets was found to be 0.45% indicating sufficient physical strength for the prepared formulations. The particle size distribution of PPF pellets represented a narrow range of 650–1100 µm with an average particle size of 950 µm. The measured bulk density for selected formulation was 0.588 g/mL and tapped density 0.645 g/mL. The angle of repose θ was 24.5° (lower than 25°) indicating good flow potential for PPF pellets. This result conforms to the findings of the report by Gupta *et al.* [17]. Therefore, the pellets fell into an acceptable range for flowability properties [18].

### 2.2. In Vitro Release

**Figure 2:**
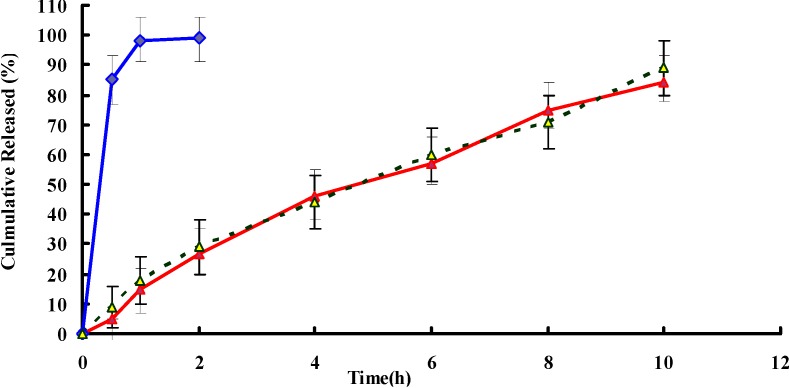
Drug release profiles of PPF pellets (red), tablets (blue) and stability samples (yellow and dotted line) in 0.1 N hydrochloric acid solution (pH 1.2) for 2 h and followed by addition of PBS solution (pH 6.8) for remaining time (*n* = 6).

*In vitro* release studies were carried out up to 12 h for all the formulations and the results have been shown in Figure 2. A very fast release behavior of PPF was observed in tablets, whereas the cumulative release rate of PPF pellets was much slower followed by a sustained release. In the tablets group, more than 90% of the drug was released in the first hour of the dissolution process in the simulated gastric medium. Almost 100% of PPF were released in the first 2 h. In contrast, only 30% of PPF were released from pellets in the first 2 h (*p* < 0.05). PPF was gradually released from the pellets over time (~50% released in 4 h, ~70% released in 8 h and ~80% released in 10 h), suggesting that PPF was well-entrapped in MCC pellets. When poorly soluble drugs such as PPF are formulated in a mixture with MCC, the drug release would be prolonged since the solubility of drug hindered its diffusion from the non-disintegrated MCC-based pellets. Furthermore, the release of poorly water-soluble drugs from the pellets is being determined by the drug/MCC ratio in the powder mixture. The higher MCC level, the more prolonged the drug release [19]. The acquired dissolution profiles were fitted into three well known mathematical models, zero order, first order and diffusion-controlled release mechanism. The relative high correlation coefficient (*r*) values obtained from the analysis of the amount of the drug released *versus* the square root of time indicated the release followed the Higuchi model [20]. Microsoft Excel was used to determine *r*^2^ of different kinetic models.

### 2.3. Stability Studies

**Figure 3:**
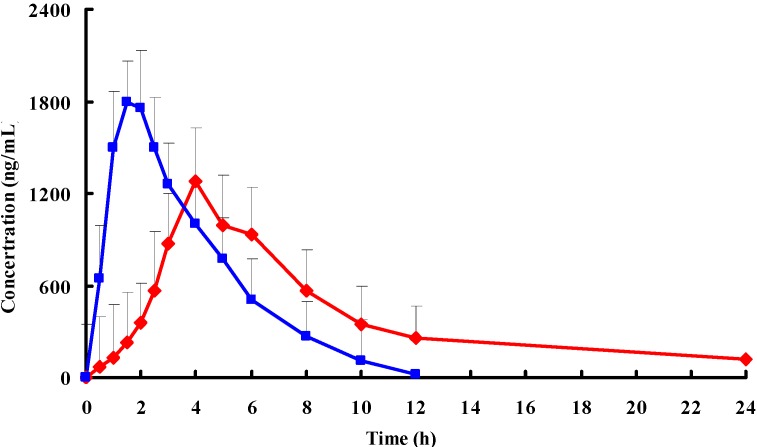
Mean plasma concentration-time profiles of PPF after oral administration of a single 20 mg/kg dose of pellets (red) and tablets (blue) to beagle dogs (each point represents the mean ± standard deviation (SD) of 6 dogs).

Stability studies were carried out for the optimized batch according to the accelerated stability study. The dissolution profile after the stability study (Figure 3) was similar compared to the samples soon after the preparation. The statistical analysis also proved sameness in dissolution profile. The stability study of pellets was made for six months at 40 °C, 75% relative humidity (RH). The dissolution profile of pellets was found to be similar, and the statistical analysis of pellets after the stability study also proved sameness (*f* = 85.8) with the pellets before the stability study. The testing of all the evaluation parameters related to the pellets were performed (data not shown). Stability study results were compared and it was found that there were no significant changes in the respective data before the stability study.

### 2.4. In Vivo Study

Pharmacokinetic studies were carried out in dogs using the PPF pellets prepared from MCC. The time course of the plasma concentrations of PPF pellets and tablets were summarized in Figure 3. The pharmacokinetic parameters calculated from the plasma drug concentration *versus* time profiles were listed in Table 1. There were significant differences in *t*_1/2_, *C*_max_, *AUC*_0–t_, *AUC*_0–∞_, MRT and *CL* between the PPF pellets and tablets (*p* < 0.05). Note: *C*_max_, maximum plasma concentration; *AUC*_0–t_, area under the concentration–time curve from time 0 to the last measurable concentration; *AUC*_0–∞_, area under the concentration–time curve extrapolated to infinity; MRT, mean residence time; *CL*, plasma clearance.

As shown in Figure 3, drug plasma concentration attained a higher level (more than 1800 ng/mL) in the initial 2 h after oral administration of PPF tablets. This corresponded with the drug’s initial burst release from tablets. The drug concentration then decreased markedly after 2 h and kept at a lower level for 6 h. Drug plasma was below the detection limit after the time of 12 h. In contrast, pellets altered the distribution of PPF *in vivo* and the half-life after oral administration was prolonged remarkably compared to those of tablets (5.72 *versus* 1.67). The result indicated that the pellets sustained release efficacy. The *AUC*_0–∞_ of the pellet formulation was 1.2-fold higher than that of tablets, suggesting that the encapsulated PPF had almost been absorbed in dogs over the period of 2 days.

**Table 1:** Pharmacokinetic parameters of the two formulations.

Parameter	Formulations	
	Tablets	Pellets
*t*_1/2_ (h)	1.67 ± 0.7	5.72 ± 2.1 ^Δ^
*C*_max_ (ng/mL)	1864.2 ± 354.6	1288.3 ± 386.6 ^Δ^
*AUC*_0–t_ (ng·h/mL)	7863.7 ± 446.3	9337.5 ± 463.1 ^Δ^
*AUC*_0–∞_ (ng·h/mL)	8272.4 ± 476.4	9891.7 ± 479.6 ^Δ^
*MRT* (h)	1.28 ± 4.6	3.82 ± 3.9 ^Δ^
*CL* (L/h)	4.54 ± 2.7	1.33 ± 2.4 ^Δ^

^Δ^ *p* < 0.05: PPF pellets *versus* PPF tablets.

## 3. Materials and Methods

### 3.1. Materials

PPF and PPF tablets were procured as a gift sample from Comb. Ltd., Hubei, China. Eudragit S100 was procured as a gift sample from Rohm Pharma (Shanghai, China), Hydroxy Propyl Methyl Cellulose (HPMC), lactose and microcrystalline cellulose (MCC) were commercially purchased from FMC (Shanghai, China). All other reagents and chemicals used were of analytical grade.

### 3.2. Preparation of Propafenone Hydrochloride (PPF) Pellets

The pellets of PPF were prepared by extrusion and spheronization technique. PPF (25%), lactose (22.5%), HPMC (4.5%) and MCC (48%) were passed through sieve No. 40 prior to pelletization and mixed uniformly. The appropriate quantity of water was then added gradually while the wetted mass was mixed for 30 min to ensure entrapment of the drug into the MCC. The obtained dough mass was extruded using a piston extruder (1.0 mm orifice) and a length of 6 mm at a constant extrusion rate of 300 rpm. The rod-shape extrudates were immediately transferred to a spheroniser equipped with a 22.5 cm radial cut plate and spheronised at 1000 rpm for 5 min. The prepared pellets were then dried in a desiccator containing silica gel at 40 °C for 6 h to guarantee an optimum and uniform level of humidity into the pellets [10,21]. The obtained pellets were coated with Eudragit S100 (5% *w*/*v*) ethanolic solutions in a fluidized bed coater (Midi-Glatt, Berlin, Germany). The process parameters were as follows: inlet temperature, 40 °C; product temperature, 30 °C; spray rate, 1.0 mL/min; atomization pressure, 0.1 MPa; batch size, 300 g; nozzle diameter, 1.0 mm.

### 3.3. Analysis of Drug Content

Around 1 g of PPF pellets was placed in a mortar, and then grinded into fine powder using a pestle. One hundred milligrams of resulting powder were accurately weighed and dissolved in 50 mL of methanol. One milliliter of the above solution was then diluted to 25 mL, filtered through 0.45 mm Millipore filters (Merck KGaA, Darmstadt, Germany). The PPF concentration in the filtrate was determined by a HPLC method described as below.

### 3.4. Characteristics of the Pellets

The particle size distribution and average diameter of the pellets were evaluated by conventional sieve analysis method using 16, 20, 25 and 30 mesh screens.

The friability test indicates mechanical strength and physical stability of the pellets [21]. Ten grams of pellets were placed in a friability test apparatus (CJY-300B, KangPu, Hunan, China) and rotated at 25 rpm for 4 min. Then the pellets were removed, de-dusted and accurately weighed to calculate the percentage weight loss.

The bulk density (*V*_b_) was determined by filling 50 g pellets into a graduated cylinder and calculating the ratio of the sample weight to sample volume. The tapped density (*V*_t_) was determined as the ratio of the sample weight to the final sample volume [22].

The angle of repose (θ) indicates flow ability of powders, granules and pellets. It was defined as the angle formed between the horizontal plane and the surface of the powder heap. To measure the angle of repose, pellets were allowed to flow through the funnel onto the graph paper. The radius (*r*) and the height (*h*) of the cone formed on the paper were measured and the angle was calculated using the following equation [23]. Tan θ = *h*/*r*.

### 3.5. In Vitro Release Studies

The release studies of the drug from the PPF pellets were carried out according to the USP dissolution II paddle method at a rotation speed of 50 rpm in 900 mL of dissolution medium at 37 ± 0.5 °C using a DST-600A dissolution tester. The release studies were also carried out at pH 1.2 medium (HCL solution) for 2 h followed by pH 6.8 medium (PBS solution) for 8 h to simulate the *in vivo* conditions. The samples were withdrawn at predetermined time intervals, and PPF was assayed by an HPLC method described below after filtration through 0.45 mm Millipore filters. The samples of accelerated storage condition (40 °C, 75% RH) for 6 months were also investigated with *in vitro* release.

### 3.6. Stability Study

Stability studies of the optimized batch were carried out by packing the pellets into a suitable packaging and subjecting to an accelerated stability study at 40 °C, 75% RH conditions as per International Conference on Harmonisation (ICH) guidelines for a period of six months. The pellets were then analyzed for different evaluation parameters (e.g., flowability, strength, shape factor, and drug release).

### 3.7. HPLC Analysis

HPLC analysis was performed using a Dikma Diamonsil C_18_ (5 μm, 150 × 4.6 mm; Dikma, Beijing, China) on a Shimadzu^®^ HPLC system (LC-20A; Shimadzu Corporation, Tokyo, Japan) with an ultraviolet detector at room temperature. The wavelength of the ultraviolet detector was set at 220 nm. Methanol, acetonitrile and 0.05 mol/L KH_2_PO_4_ solution (30:20:50, *v*/*v*/*v*) was used as the mobile phase at a flow rate of 1 mL/min.

A 500 µL volume of the plasma sample was transferred to a 5 mL plastic test tube together with 10 µL of internal standard (I.S.) solution (2 mg/mL chlorpromazine). After vortex shaking for 1 min (5432 vortex mixer, Eppendorf, Hamburger, Germany), 2 mL of diethyl ether was added and the mixture was vortexed for 3 min. After centrifugation at 12,000 rpm for 10 min (Thermo IEC, Micromax, Boston, MA, USA), the upper organic layer was quantitatively transferred to a 10-mL glass tube and evaporated to dryness using an evaporator at 40 °C. The residue was reconstituted in 100 µL of the mobile phase, and then vortex-mixed. After centrifugation at 12,000 rpm for 5 min, a 10 µL aliquot of the solution was injected into the HPLC system for analysis.

### 3.8. Pharmacokinetic Study

#### 3.8.1. Animals

Six beagle dogs (weight: 9–11 kg) were supplied by the Hospital Animal Center (Shanghai, China). Prior to use, all dogs were maintained under standard laboratory conditions on a 12 h light/dark cycle and were fed standard chow and sterilized tap water. All experimental procedures were carried out in accordance with the guidelines of the Animal Care Committee of the Hospital Laboratory Animal Center and approved by the center (Register ID No.: AX05128; 1 April 2013).

#### 3.8.2. Study Design

The study was carried out as a two-period, balanced, randomized cross-over investigation in which the PPF pellets and tablets were given in a single dose of 20 mg/kg with 50 mL water by oral administration. Six dogs were randomly assigned to one of the two groups taking either the test preparation or the reference preparation. After a seven-day washout period the subjects were cross-overed.

#### 3.8.3. Blood Sampling

Blood samples were collected in heparinized tubes prior to the drug administration-zero time, and at 0.25, 0.75, 1, 1.5, 2, 3, 4, 6, 8, 10, 12, 24 h after dosing. Blood samples (2.5 mL) were centrifuged (3000 rpm/10 min) and the plasma collected was stored at −18 °C until analysis. Pharmacokinetic parameters were calculated from the plasma concentration-time data. The elimination half-life (*t*_1/2_) was determined by linear regression of the terminal portion of the plasma concentration-time data. The area under the plasma concentration-time curve from zero to the last measurable plasma concentration point (*AUC*_0–t_) was calculated by the linear trapezoidal method. Extrapolation to time infinity (*AUC*_0–∞_) was calculated as follows: *AUC*_0–∞_ = *AUC*_0–t_ + *C*_t_/*k*_e_, where *C*_t_ is the last measurable plasma concentration and *k*_e_ is the terminal elimination rate constant. The results were expressed as mean ± standard deviation (SD).

#### 3.8.4. Statistical Analysis

Values were expressed as mean ± SD for each group. Statistical evaluation of the experimental data was performed using Student’s *t*-test. A *p* < 0.05 was considered statistically significant.

## 4. Conclusions

An extrusion–spheronization method was successfully applied to fabricate PPF sustained-release pellets. Using scanning electron microscopy, it was shown that the PPF pellets had a mean size of approximately 950 µm with a spherical shape. The *in vitro* release profiles indicated that the release of PPF from the pellets exhibited a sustained release behavior. A similar phenomenon was also observed in a pharmacokinetic study in dogs, in which the AUCs of the pellet formulations were 1.2-fold higher than that of PPF tablets. The present work demonstrates the feasibility of controlled delivery of PPF utilizing MCC-based pellets.
